# Chemopreventive effects of Xiang Sha Liu Jun Zi Tang on paclitaxel-induced leucopenia and neuropathy in animals

**DOI:** 10.3389/fphar.2023.1106030

**Published:** 2023-03-02

**Authors:** Her-Shyong Shiah, Chia-Jung Lee, Fang-Yu Lee, Sung-Hui Tseng, Shih-Han Chen, Ching-Chiung Wang

**Affiliations:** ^1^ Division of Hematology and Oncology, Department of Internal Medicine, Taipei Medical University Hospital, Taipei, Taiwan; ^2^ Graduate Institute of Cancer Biology and Drug Discovery, College of Medical Science and Technology, Taipei Medical University, Taipei, Taiwan; ^3^ Division of Hematology and Oncology, Department of Internal Medicine, Taipei Tzu Chi Hospital, Buddhist Tzu Chi Medical Foundation, New Taipei, Taiwan; ^4^ Traditional Herbal Medicine Research Center, Taipei Medical University Hospital, Taipei, Taiwan; ^5^ Graduate Institute of Pharmacognosy, Taipei Medical University, Taipei, Taiwan; ^6^ Ph.D. Program in Clinical Drug Development of Herbal Medicine, College of Pharmacy, Taipei Medical University, Taipei, Taiwan; ^7^ Department of Physical Medicine and Rehabilitation, Taipei Medical University Hospital, Taipei, Taiwan; ^8^ School of Pharmacy, Taipei Medical University, Taipei, Taiwan

**Keywords:** paclitaxel, national health insurance research database (NHIRD), xiang sha liu Jun zi tang, leucopenia, neuropathy, dorsal root ganglion cells

## Abstract

Paclitaxel frequently induces peripheral neuropathy and myelosuppression during cancer treatment. According to the National Health Insurance Research Database of Taiwan, traditional Chinese medicine doctors widely use Xiang Sha Liu Jun Zi Tang (XSLJZT) to treat breast cancer patients who have received paclitaxel. We explored the combined therapeutic effects of XSLZJT with paclitaxel. XSLJZT did not exhibit significant cytotoxic effects on P388-D1 cells; however, the combination of XSLJZT (100 and 500 mg/kg) with paclitaxel prolonged the survival rate in P388–D1 tumor-bearing mice compared to paclitaxel-only. In addition, XSLJZT was found to enhance white blood cells (WBC) counts and promote leukocyte rebound in paclitaxel-induced leukopenia in mice. XSLJZT also reduced paclitaxel-induced mechanical pain and inhibited c-Fos protein expression in the L4-6 spinal cords of Wistar rats. Moreover, paclitaxel-induced shortening of the nerve fibers of dorsal root ganglion cells was ameliorated by pre-treatment with XSLJZT. Therefore, we suggest that XSLJZT could be used as an adjunct for cancer patients, as the formula could decrease paclitaxel-induced neuropathy and myelosuppression.

## 1 Introduction

Breast cancer is the most common cancer in women. Paclitaxel, a taxane agent, is a widely used chemotherapeutic drug to treat breast cancer. Paclitaxel is a microtubule inhibitor that can inhibit mitosis in cancer cells. It is used in hormone-negative but HER-2 positive, hormone-negative, and HER-2 negative (triple negative) breast cancer patients (Network, 2022). Pancytopenia, hair loss, arthralgia, myalgia, peripheral neuropathy, nausea and vomiting, and diarrhea remain some of the common side effects of paclitaxel treatment (Rowinsky and Donehower, 1995; Drake et al., 1998). Paclitaxel-induced peripheral neuropathy ranges from mild paresthesia to severe neuropathic pain (Scripture et al., 2006). Neutropenia is another principal dose-limiting toxicity of paclitaxel. Typically, paclitaxel-related toxicity is managed by dose delay, reduction, or discontinuation, which may affect the patient’s overall survival. These symptoms and signs affect the quality of life of patients undergoing chemotherapy (Kirca and Kutluturkan, 2018). Therefore, agents that can prevent or ameliorate paclitaxel-related toxicity will help patients complete the scheduled treatment course. Complementary therapies (CTs) have shown beneficial effects on cancer patients for symptomatic relief and have increased their quality of life (Satija and Bhatnagar, 2017). Chinese herbal medicine (CHM) is one of the most frequently used CTs to help control chemotherapy-induced symptoms such as nausea and vomiting, pain, and fatigue (Lv et al., 2018).

In Taiwan, the incidence of breast cancer increased by 1.8-fold between 1997 and 2013 (Liu et al., 2017). Breast cancer patients who used CHM for >30 days had a significantly higher survival probability than non-CHM users (Lee et al., 2014). Among the co-prescriptions of CHM and chemotherapeutic agents, Xiang Sha Liu Jun Zi Tang (XSLJZT) is one of the most frequently used formulas to alleviate the symptoms associated with breast cancer treatment (Wang et al., 2014).

XSLJZT, originating from the classic book “Yizong Jinjian,” has been used clinically since the Qing Dynasty in 1742. In traditional Chinese Medicine pharmacology, XSLJZT can invigorate the spleen, harmonize the stomach, and regulate the Qi flow to relieve pain. Studies have shown that XSLJST is safe and effective for treating functional dyspepsia (Xiao et al., 2012). A randomized, double-blind, placebo-controlled study also provides evidence supporting the effectiveness of XSLJZT in improving gastric symptoms of irritable bowel syndrome (Shih et al., 2019). Liu Jun Zi Tang (LJZT) is an XSLJZT-related formula used to tonify Qi, invigorate the spleen, and remove phlegm. Both formulas contain the following herbs at different concentrations: *Panax ginseng* C. A. Mey*er* (ginseng), *Atractylodes macrocephala* Koidz (atractylodes), *Wolfiporia extensa* (Peck) Ginns (hoelen), *Glycyrrhiza uralensis* Fisch*.* ex DC. (licorice), *Citrus × aurantium f. Deliciosa* (Ten.) M. Hiroe (citrus), *Pinellia ternata* (Thunb.) Makino (pinellia) and *Zingiber officinale* Roscoe **(**ginger). XSLJZT includes two more herbs: *Wurfbainia longiligularis*. (T.L.Wu). Skornick. and A.D.Poulsen (cardamom), and *Dolomiaea souliei* (Franch.) C. Shih (saussurea). Preclinical studies indicate that LJZT attenuates cisplatin-induced neurotoxicity (Chiou et al., 2018). However, the potential benefits of XSLJZT in preventing and treating paclitaxel-induced side effects remain unclear. This study aimed to explore the effectiveness of XSLJZT against paclitaxel-induced neurotoxicity and leukopenia.

## 2 Materials and methods

### 2.1 Materials

Culture medium materials, including Ham’s F12 medium, Dulbecco’s modified Eagle medium (DMEM), fetal bovine serum, and penicillin/streptomycin, were purchased from GIBCO (Carlsbad, CA, United States). Isoflurane was procured from Aesica Queenborough Limited (UK). The anti-c-Fos antibody was purchased from Santa Cruz Biotechnology (CA, United States).

### 2.2 Preparation of XSLZJT

The prescription of XSLZJT was based on the unified formula announced by the Committee on Chinese Medicine and Pharmacy of the Department of Health (Taipei, Taiwan). *Panax ginseng* C. A. Mey*er, Atractylodes macrocephala* Koidz*, Wolfiporia extensa* (Peck) Ginns*, Glycyrrhiza uralensis* Fisch*.* ex DC.*, Citrus × aurantium f. Deliciosa* (Ten.) M. Hiroe*, Pinellia ternata* (Thunb.) Makino*, Dolomiaea souliei* (Franch.) C. Shih, *Wurfbainia longiligularis*. (T.L.Wu) Skornick. and A.D.Poulsen and *Zingiber officinale* Roscoe were purchased from Sun Tan Pharmaceutical (New Taipei City, Taiwan). The medicinal materials were authenticated by a non-profit organization, the Brion Research Institute of Taiwan (New Taipei City, Taiwan). Its daily dosage was shown in Table 1. Specifically, XSLZJT was immersed in a 20-fold quantity of distilled water and boiled in a herb-extracting machine until half of the original water volume remained. The extract was filtered and freeze-dried. The freeze-dried sample powder was stored at −20°C until further use.

**TABLE 1:** The prescription and phytochemical compositions of the Xiang Sha Liu Jun Zi Tang water extract.

Item	XSLJZT
	*Atractylodes macrocephala* Koidz (5 g)
	*Citrus × aurantium f. Deliciosa* (Ten.) M.Hiroe (2 g)
	*Dolomiaea souliei* (Franch.) C.Shih (2 g)
The prescription (daily dosage)	*Glycyrrhiza uralensis* Fisch ex DC. (2 g)
	*Panax ginseng* C. A. Mey*er* (2.5 g)
	*Wurfbainia longiligularis.* (T.L.Wu) Skornick. and A.D.Poulsen. (2 g)
	*Zingiber officinale* Roscoe (5 g)
Yield	25.6%
Total polyphenol	14.55 ± 0.14 mg/g
Total polysaccharide	0.88 ± 0.27 mg/g

### 2.3 Composition fingerprint analysis by UP-High performance liquid chromatography triple-quadrupole mass spectrometer

An Agilent 6,470 triple-quadrupole mass spectrometer (Agilent Technologies, CA, United States) equipped with an Agilent Jet Stream electrospray ionization (ESI) source and integrated into the UPLC system Agilent 1,290 Infinity II LC system (Multisampler: G7167B; Binary Pump: G7120A; Column: Agilent RRHD SB-C18, 2.1 × 100 mm, 1.8 μm; Column Comp: G7116B; DAD: G7111A) (Agilent Technologies, CA, United States) was used in this study. The mass spectrometer ion source settings were as follows: Gas Temp, 300 (°C); Gas Flow, 5 (L/min); Nebulizer, 45 (psi); Sheath Gas Heater, 250 (°C); Sheath Gas Flow, 11 (L/min); Capillary ESI+, 4,000 (V); ESI-, 3,500 (V); VCharging, 500 (V). UP-HPLC profile of XSLZJT was analyzed with a mobile-phase gradient elution condition: Water was used for mobile-phase A, and methanol containing 0.1% FA for mobile-phase B. The elution gradient was maintained as follows: 0–20 min, 100% A to 80% A; 20–25 min, 80% A; 25.01–40 min, 80% A to 70% A; 40.01–50 min, 70% A to 60% A; 50.01–60 min, 60% A to 50% A; 60.01–60.10 min, 60% A to 0% A; 60.10–63 min, 0% A. The sample was analyzed using a UV/Vis spectrophotometer at 280 nm. Multiple reaction monitoring (MRM) mode was used during mass spectrometry to detect hesperidin, atractylenoide III, and glycyrrhizic acid. The ion pairs were ESI- m/z 609.3→301.1 (Frag 198 V, CE 26 V), ESI+ 249.1→231.1 (Frag 132 V, CE 10 V), ESI- 821.1→351. (Frag: 206 V; CE: 46 V).

### 2.4 Total polyphenol analysis

The total phenol content was determined using the Folin-Ciocalteau method (Tseng et al., 2006). The XSLZJT aqueous extract was dissolved in double-distilled (dd)H_2_O. The sample solution was mixed with Folin–Ciocalteu reagent and a 7.5% aqueous Na_2_CO_3_ solution. After 5 min incubation at 50°C, the absorbance was measured at 600 nm against water using an enzyme-linked immunosorbent assay (ELISA) reader. The total phenol content was expressed as gallic acid equivalents (mg GA/g sample) using a calibration curve prepared from gallic acid standard concentrations (15.625–250 μg/mL) and was fitted with the following equation: y = 0.00494x − 0.105 (*r* ^2^ = 0.991).

### 2.5 Total polysaccharide analysis

The total polysaccharide content was determined using the phenol-sulfuric method (Yu et al., 2017). The XSLZJT solution was mixed with 95% ethanol. After being allowed to stand for 30 min at room temperature, the precipitate was collected, and a 5% phenol solution and 2 M sulfuric acid were added. The well-mixed solution was shaken for 30 min, and its absorption was measured at 485 nm against water using an ELISA reader. The amount of total polysaccharide was expressed as glucose equivalents (mg glucose/g sample) using a calibration curve prepared from standard amounts of gallic acid of 6.25∼100 μg/mL and was fitted with the following equation: y = 2.4177x − 0.0097 (*r* ^2^ = 0.997).

### 2.6 Animals

CDF1 mice (DBA male × BALB/c female), BALB/c-nu mice 20 ± 5 g), and Wistar Rats 200 ± 20 g) were purchased from BioLASCO Taiwan Co., Ltd. (Taipei, Taiwan), housed in a controlled environment at 21°C with sufficient food and water, and kept in an alternating 12 h dark and light cycle. The animal experiments were approved by the Ethical Regulations on Animal Research of Taipei Medical University (Approval No: LAC-2014-0372).

### 2.7 Cell viability and survival rates of XSLJZT and paclitaxel in P388-D1 and P388-D1 tumor-bearing CD2F1 mice

P388-D1 cells of a mouse macrophage lymphoma cell line were cultured in DMEM with 10% fetal bovine serum (FBS), 1% penicillin-streptomycin, and 1% L-glutamine at 37°C in a 5% CO_2_ atmosphere. P388-D1 cells (10^5^ cells/well) were seeded in 24-well plates and co-treated with or without paclitaxel, and the test samples were incubated for 24 h. Cell proliferative activity was detected using a 3-(4,5-dimethylthiazol-2-yl)-2,5-diphenyltetrazolium bromide (MTT) assay.

P388-D1 cells (10^6^ cells/mouse) were intraperitoneally (*i.p*.) transplanted into 5-week-old female CDF1 mice. The animals were allowed to rest for 24 h before treatment began. The control group was administered distilled water for the entire process. The XSLJZT group was orally administered XSLJZT (500 mg/kg/day) during the entire process. The paclitaxel group (20 mg/kg) received paclitaxel intraperitoneally on days 1, 3, 5, 7, and 9. Paclitaxel (20 mg/kg) and XSLJZT co-administered (100 or 500 mg/kg) group was performed in the order of *i.p* administration of paclitaxel (day 1, day 3, day 5, day 7, and day 9) and immediately oral administration of XSLJZT (the whole process).

The number of days the animal survived was observed and recorded until the day the animal died. There were eight mice in each group. The antitumor effect was defined as the percent increase in the life span (%ILS) calculated according to the following equation: %ILS = [(T/C)–1] × 100%, where T and C respectively represent the mean survival times (days) of the treated and vehicle control groups. The body weight (BW) of each CD2F1 mouse was determined daily on an animal scale. Data are presented as mean ± standard deviation (SD). The Student's t-test was used to compare survival times (days) between the test and blank groups.

### 2.8 Paclitaxel-induced hyperalgesia in Wistar Rats

We also designed pre-treatment and post-treatment groups of XSLZJT in the hyperalgesia animal model (Figure 3A). Each group consisted of five animals. In the pre-treatment groups, Wistar Rats were orally administered XSLZJT 50 or 250 mg/kg BW once a day for 15 days, followed by *i.p.* Administration of paclitaxel 2 mg/kg once on days 8, 10, 12, and 14, respectively. On day 15, each group was subjected to the Von Frey test for mechanical allodynia. In the post-treatment groups, after *i.p.* Administration of paclitaxel 2 mg/kg four times, rats were orally administered XSLZJT once a day for 9 days beginning on day 15. On day 23, each group was examined using the Von Frey test for mechanical allodynia and anesthetized with isoflurane to obtain the L4-6 spinal cords.

### 2.9 Paclitaxel-induced leukopenia in BALB/c mice

XSLZJT pre-treatment and post-treatment were designed for paclitaxel-induced leukopenia in a BALB/c mouse model (Figure 3B). BALB/c mice were divided into blank, induced, and XSLZJT groups, with each group containing six mice. In the pre-treatment groups, BALB/c mice were orally administered XSLZJT at a dose of 500 mg/kg BW once a day for 16 days and then received paclitaxel intraperitoneally (*i.p.*) at 10 and 6.8 mg/kg doses once on days 8 and 14, respectively. In the post-treatment groups, BALB/c mice were orally administered XSLZJT once daily for 9 days after *i.p.* Paclitaxel administration on day 8. BALB/c mice in the blank group were *intraperitoneally injected* with normal saline, and sterile water was administered orally. In the induced group, *i.p.* Paclitaxel was administered, and sterile water was administered orally. Whole blood was obtained from the eyehole vein of BALB/c mice on days 10 and 16. White blood cell (WBC) levels of the whole blood were analyzed using an automatic multi-parameter blood cell counter (Sysmex KX-21 N). The WBC levels of control and XSLJZT compared with the blank group were calculated based on the following equation: Control or XSLJZT/blank × 100%. Data are presented as mean ± SD.

### 2.10 Von Frey test for mechanical allodynia

The Von Frey test was performed as described previously (Yi et al., 2019) and assessed on days 15 (pre-treatment group) and 23 (post-treatment group). Briefly, in the thermal hyperalgesia analysis, animals were individually placed on a dynamic plantar esthesiometer (Ugo Basile, Comerio, Varese, Italy) with a mesh screen floor. A movable touch-stimulator unit was placed under the floor. The apparatus applied a Von Frey (0.5 mm) filament to the plantar surface, increasing the force incrementally (0–50 g) until it reached the paw withdrawal threshold. The device automatically recorded the force at which paw withdrawal occurred, and the withdrawal force (g) was used as an indicator to identify the effects on pain response. Data are presented as mean ± SD.

### 2.11 Histologic assessment

L4-6 spinal cords fixed in 10% (v/v) neutral buffered formalin for 24 h, paraffin-embedded, and cut into 5 μm thicknesses for histopathological assessment (Li et al., 2021). c-Fos immunostaining was performed on the paraffin-embedded sections. After staining, the pathological changes in the dorsal horn ganglion were observed under a light microscope. c-Fos would be stained brown, and the color intensity depended on c-Fos expression.

### 2.12 Cell viability of primary culture dorsal root ganglion (DRG) cells assay

Six-week-old male Wistar Rats were anesthetized with isoflurane, the spinal cord was removed, the DRG was harvested, and was cleaned under a stereoscopic microscope. DRG were dissociated into single cells using a two-step enzyme treatment. First, chopped DRG was treated with 0.125% collagenase type I at 37°C for 90 min with gentle shaking. The cell pellet was collected by centrifugation at 1,500 × g for 5 min. Second, the cell pellet was incubated with 0.25% trypsin-EDTA solution at 37°C for 30 min and triturated with fire-polished Pasteur pipettes with decreasing tip diameter. Next, the cells were plated on poly-D-lysine (PDL)-coated 8-well chamber slides and 24-well microtiter culture plates incubated in Ham’s F12 medium containing 10% heat-inactivated fetal bovine serum, followed by 1% penicillin/streptomycin for up to 5 days. The cultures were maintained at 37°C in a humidified atmosphere containing 5% CO_2_.

A stock solution of XSLJZT (10 mg/mL) was prepared by dissolving the test samples in ddH2O and storing the mixture at 4°C until further use. On the 6th day of culture, test samples at the appropriate concentrations were added to the culture plates for 24 h without changing the medium in serum-free medium with NGF (10 ng/mL). The number of surviving cells in 24-well microtiter culture plates was counted using the tetrazolium (MTT) assay (Yu et al., 2017). The cytotoxicity index (CI, %) was calculated based on the following equation: CI = [1 − (T/C)] × 100%, where T and C respectively represent the mean optical densities of the treated and vehicle control groups.

### 2.13 Measure the length of the axonal branch of the DRG cell

The DRG cells, treated with or without test samples on 8-well chamber slides, were fixed with 4% paraformaldehyde in 0.1 M phosphate buffer (pH 7.4) for 20 min at room temperature permeabilized with PBS containing 0.2% Triton X-100 and blocked with goat serum and BSA. Next, the cells were immunostained with a polyclonal antibody against peripherin (dilution 1:1,000) as a sensory nerve marker. Alexa Fluor 488-conjugated goat anti-rabbit IgG dilution (1:200) was used as the secondary antibody. The slides were mounted using Mount Quick Mounting Medium. Fluorescent images (ten images per treatment) were captured using a fluorescence microscope (DMI 4000 B, Leica). The length of neurites positive for peripherin was measured using ImageJ software (Rasband, W.S., Image J, U.S. National Institutes of Health, Bethesda, Maryland, United States). At least 15 cells from triplicate wells per group were analyzed for each experiment.

### 2.14 Statistical analysis

Results are presented as mean ± standard deviation (SD). Data were analyzed using the SPSS software (version 17.0; SPSS Statistics for Windows, Chicago, IL, United States). Group differences were statistically assessed by one-way analysis of variance (ANOVA), followed by Fisher’s least significant difference (LSD) test to compare means. Statistical significance was set at *p* < 0.05.

## 3 Results

### 3.1 Composition and phytochemical contents of XSLJZT

**FIGURE 1:**
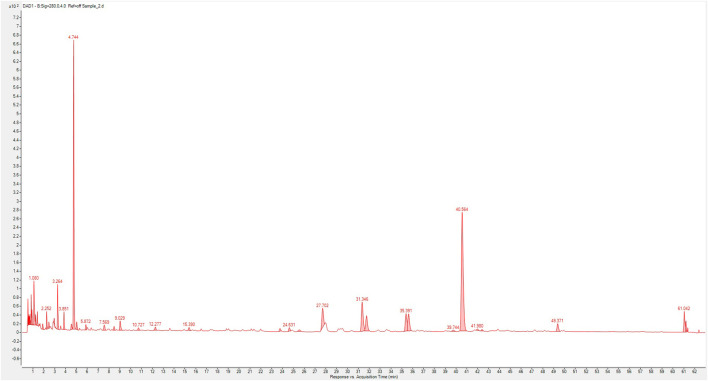
Composition fingerprint analysis of XSLZJT by UP-High Performance Liquid Chromatography triple-quadrupole mass spectrometer. (1), hesperidin; (2), atractylenoide III; (3), glycyrrhizic acid.

In this experiment, we used UP-HPLC fingerprint profile to confirm the possible components in XSLJZT. The results show that hesperidin, atractylenoide III, and glycyrrhizic acid as the major compounds in XSLJZT (Figure 1). Further, each gram of XSLJZT contained the hesperidin, glycyrrhizin, and atractylenolide III were 24.52, 11.83, and 0.24 mg, respectively. However, the low polar components, like ginsenosides, volatile oils, lactones, were not easy to extract. Hot water extraction findings suggested that the major components of the extract appear to be polar compounds. Therefore, total polyphenols and polysaccharides were used as quality indicators of XSLJZT. In XSLJZT, the total polyphenol concentration was 14.55 ± 0.14 mg/g, while the total polysaccharide concentration was 0.88 ± 0.27 mg/g.

### 3.2 Cytotoxicity and therapeutic effects of XSLJZT and paclitaxel in P388-D1 and P388-D1 tumor-bearing CD2F1 mice

**FIGURE 2:**
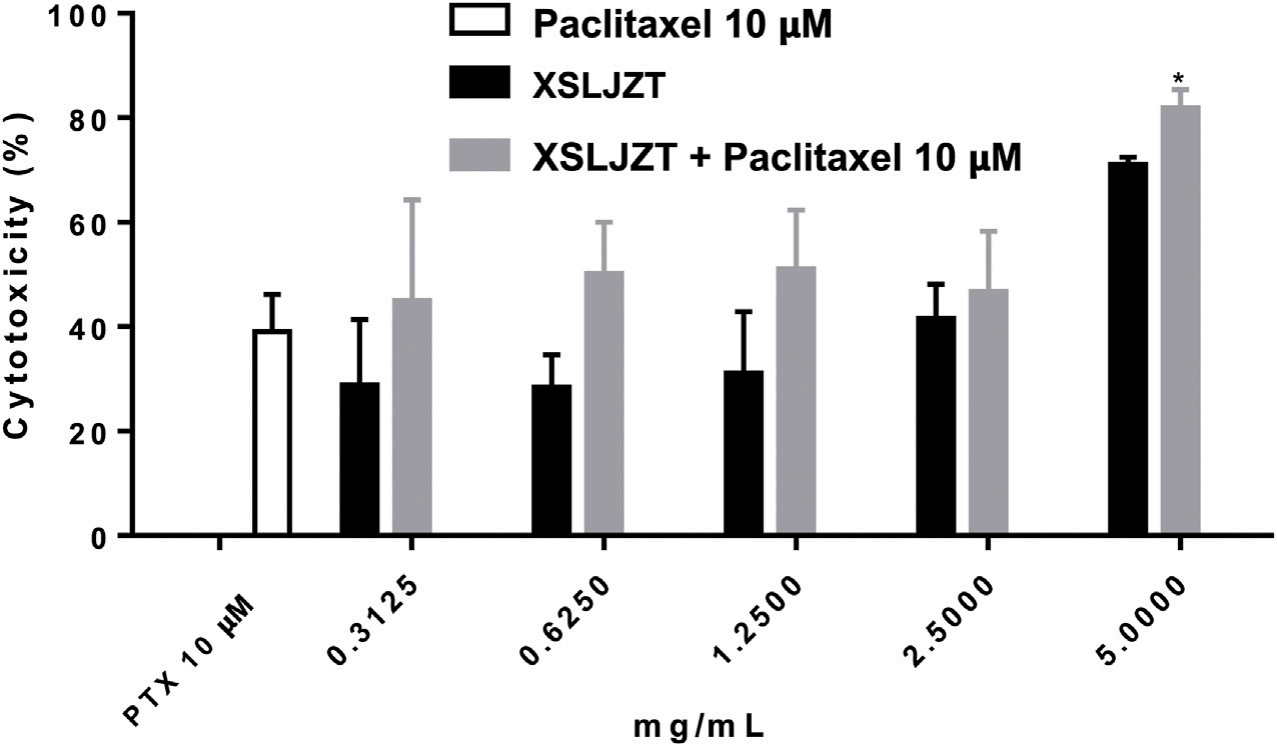
Cytotoxic effects of paclitaxel XSLJZT and XSLJZT combined with paclitaxel on P388-D1 cells. Paclitaxel significantly inhibited the growth of P388-D1 cells at 10 µM. 5 mg/mL of XSLJZT combined with paclitaxel was significantly better than paclitaxel *: *p* < 0.05, *n* = 3.

The cytotoxic effects of paclitaxel and XSLJZT were evaluated using an MTT assay. Paclitaxel was found to affect the cells in a dose-dependent manner from 2.5 − 20 µM (data not show). It significantly inhibited P388-D1 cells with a 50% inhibitory concentration (IC_50_) of 11.34 ± 0.33 µM. XSLJZT exhibited an inhibitory effect on P388-D1 cells at a 5 mg/mL concentration with an IC_50_ value of 2.98 ± 0.81 mg/mL. The P388-D1 cells were treated with paclitaxel and XSLJZT. At a high dose (5 mg/mL), XSLJZT significantly enhanced toxicity toward P388-D1 cells. (Figure 2).

The antitumor effects of paclitaxel alone and paclitaxel combined with XSLJZT were evaluated using P388-D1 cells in an *in vivo* model. P388-D1 cells were injected into the intraperitoneal cavity of CD2F1 mice to induce cancer ascites. There was an abnormal increase in the abdomen size and BW. As shown in Table 2, results demonstrated an increase of approximately 44.3% in the survival rate of the group receiving paclitaxel compared to the control group. XSLJZT alone (500 mg/kg) slightly increased the survival rate by 4%, indicating no significant *in vivo* antitumor effect of XSLJZT. The co-administration of paclitaxel and XSLJZT (100 and 500 mg/kg) significantly improved the survival rate compared to the control group by 44.9% and 55.4%, respectively. However, the combination of XSLJZT with paclitaxel increased by 11.1% compared to the group receiving paclitaxel. These results suggest that XSLJZT does not influence the *in vivo* antitumor effect of paclitaxel.

**TABLE 2:** Survival rate of P388-CD2F1 tumor-bearing mice with different treatments.

Group	Number of days for the last death	Mean survival rate	ILS%
Control	25	21 ± 3.0	—
XSLJZT (500 mg/kg)	29	22.3 ± 2.8	4.0 ± 13.2
Paclitaxel (100 mg/kg)	34	30.9 ± 2.3 *	44.3 ± 16.3
Paclitaxel (100 mg/kg) +XSLJZT (100 mg/kg)	34	31.0 ± 2.7*	44.9 ± 12.8
Paclitaxel (100 mg/kg) +XSLJZT (500 mg/kg)	38	33.3 ± 3.0 *	55.4 ± 14.0

Compared to the control group *presented as *p* < 0.05.

### 3.3 Reversal of paclitaxel-induced leucopenia by XSLJZT in mice

**FIGURE 3:**
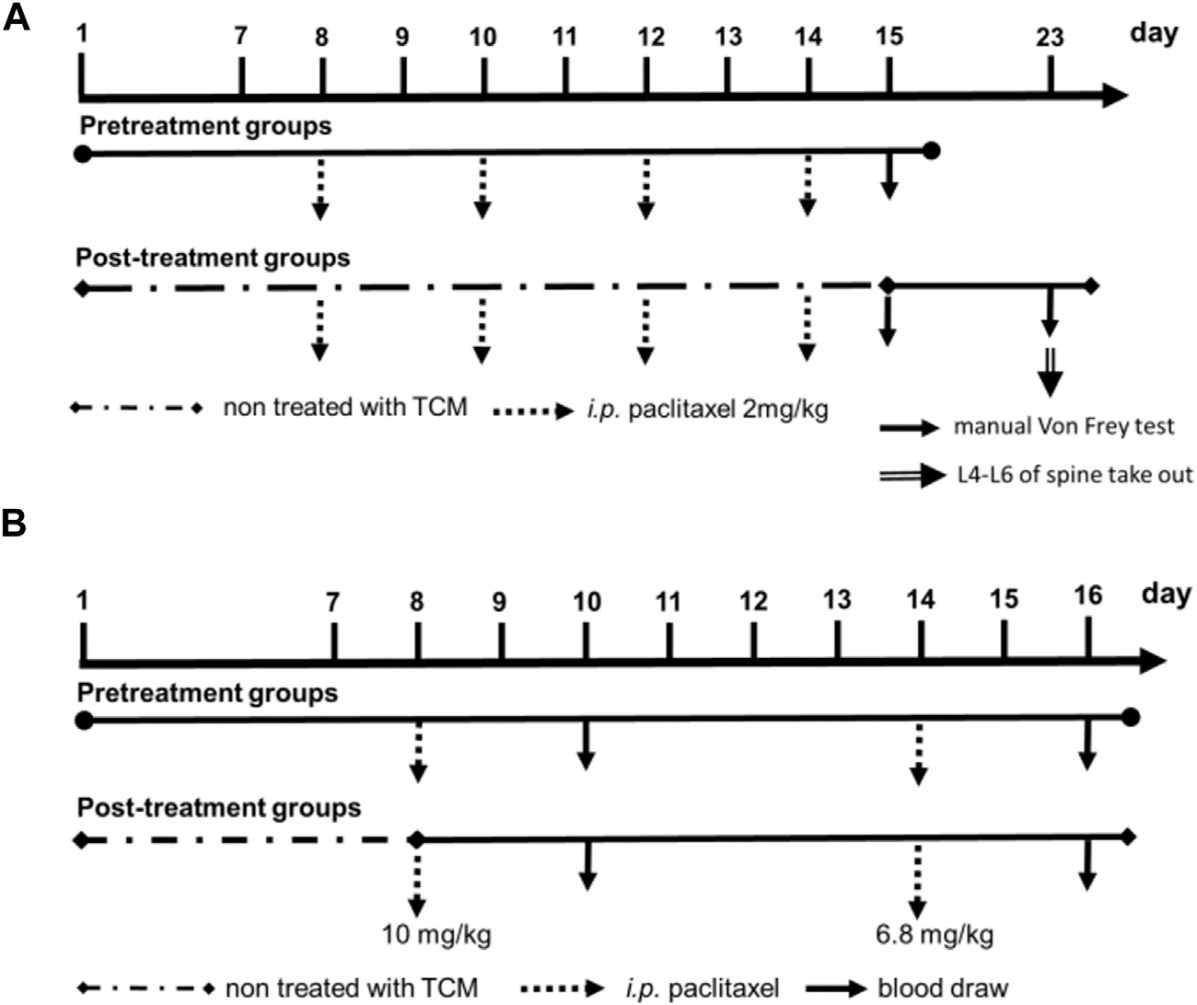
Experimental flow cart. **(A)**, paclitaxel-induced leukopenia in BALB/c mice model; **(B)**, pre-treatment and post-treatment of XSLZJT in the hyperalgesia animal model.

**FIGURE 4:**
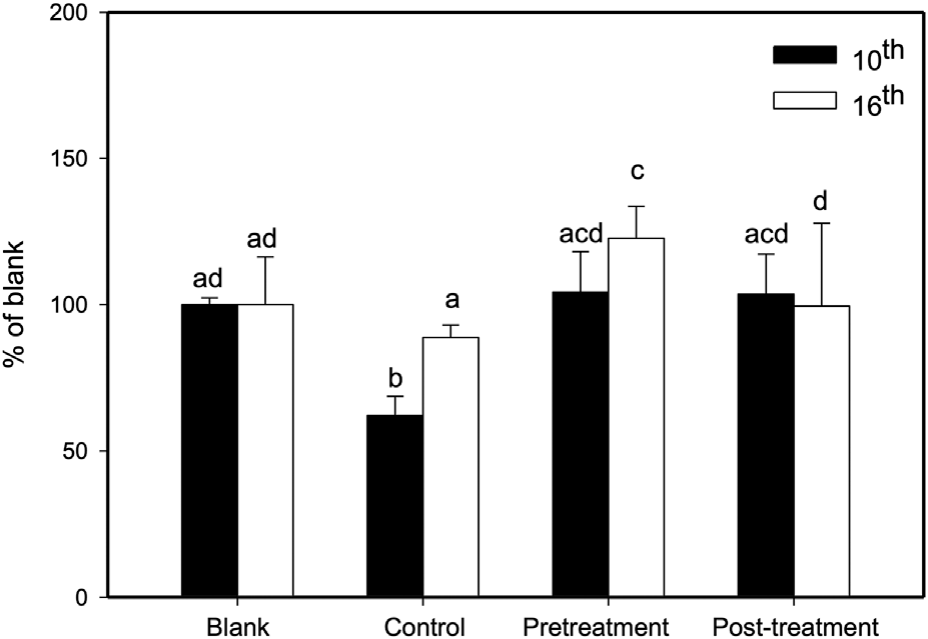
Effects of XSLJZT on paclitaxel-induced leucopenia in mice on days 10 and 16. Results are presented as the mean ± S.D. The different alphabets show a significant (*p* < 0.05) difference between each other.

We studied the preventive and treatment effects of XSLJZT on paclitaxel-induced leukopenia in mice. The study procedure is illustrated in Figure 3. Two days after the first paclitaxel injection (10 mg/kg), the no-treatment group showed significantly reduced WBC counts compared with the blank group. Administration of 500 mg/kg XSLJZT before or after paclitaxel injection reversed neutropenia 2 days later but without significant intergroup differences. On day 16, the control group (paclitaxel-only) also showed a lower WBC count, but not similar to that on day 8. However, both the pre- and post-treatment groups showed normal levels of white blood cells, with the pre-treatment group showing a higher ability to reverse paclitaxel-induced leukopenia. These results suggest that XSLJZT could be beneficial as a preventive or therapeutic agent to overcome the immunosuppression induced by paclitaxel. XSLJZT, administered before paclitaxel treatment and continued for 16 days, showed the highest white blood cell count (Figure 4).

### 3.4 Anti-mechanical allodynia effects of XSLZJT on paclitaxel-induced neuropathy in rats

**FIGURE 5:**
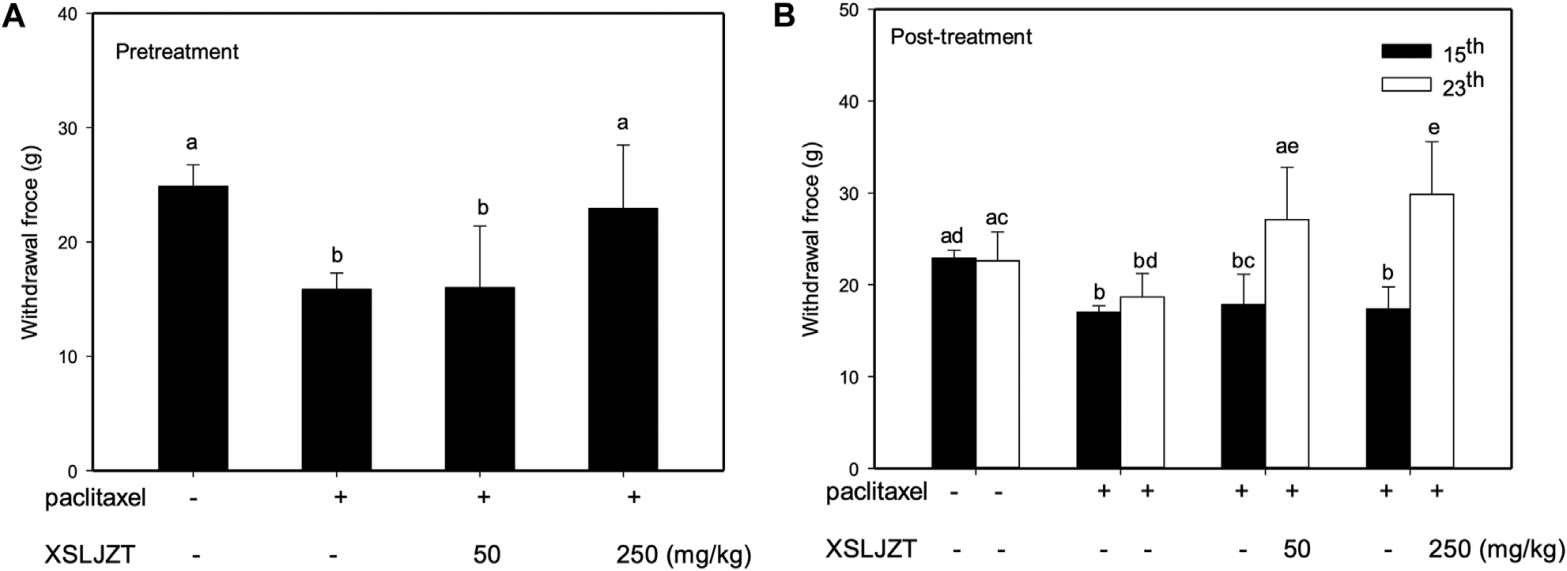
Anti-mechanical allodynia effects of XSLZJT on paclitaxel-induced neuropathy in rats. **(A)**, pre-treatment effects of XSLJZT (50 or 250 mg/kg) on the von frey test results on day 15; **(B)**, post-treatment effect of XSLJZT on the Von Frey test results on day 15 and day 23 as compared to the control group. The different alphabets show a significant (*p* < 0.05) difference between each other.

**FIGURE 6:**
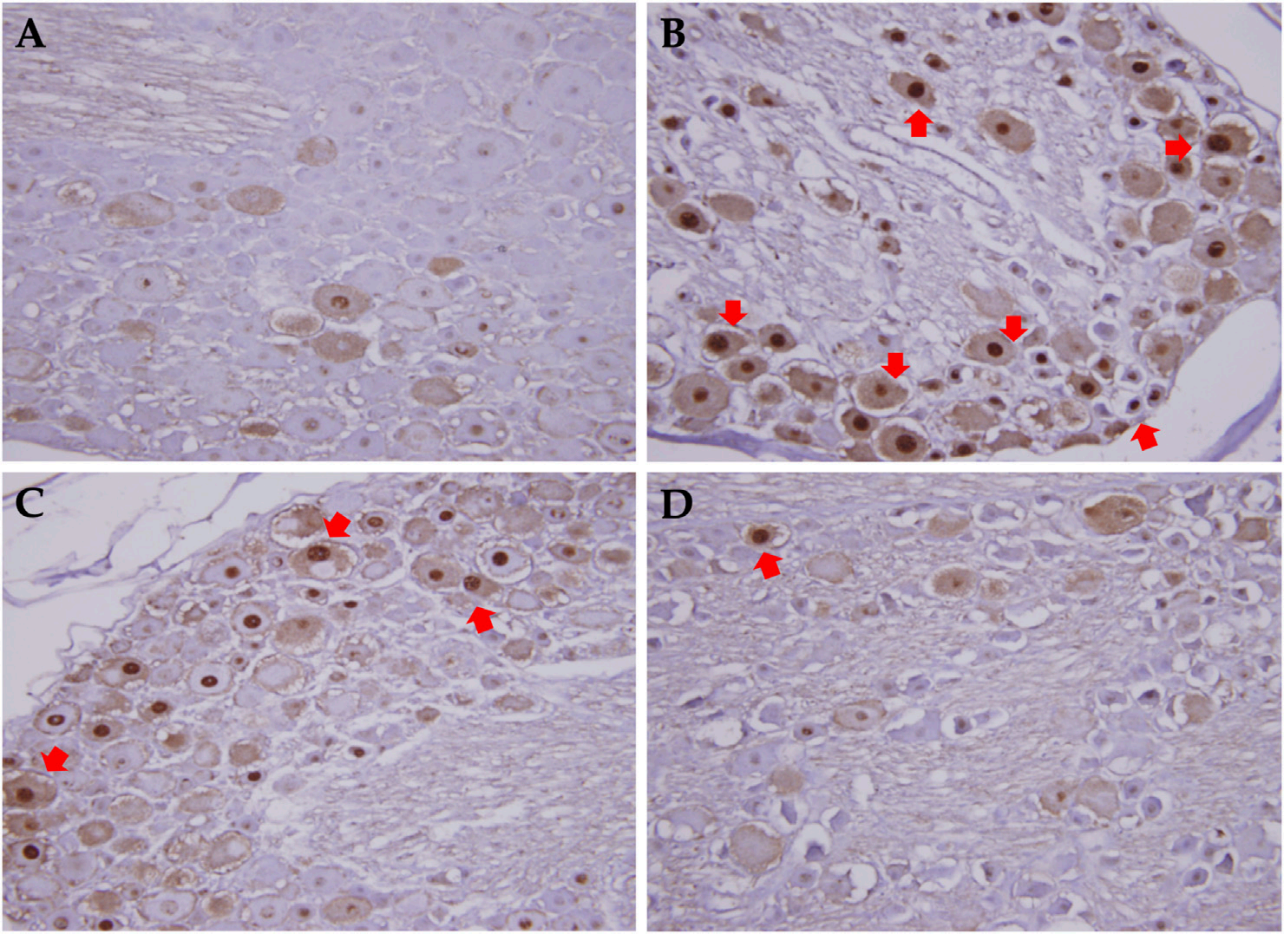
c-fos expression in dorsal root column in rats treated with or without XSLZJT pre- and post-paclitaxel treatment. **(A)**, the spinal cord of blank group showed nearly no c-fos staining; **(B)**, paclitaxel only group showed significant c-fos protein expression; **(C)**, c-fos expression in XSLJZT pre-treatment group; **(D)**, c-fos expression in XSLJZT post-treatment group.

In this study, the pre-treatment group received 50 or 250 mg/kg XSLZJT orally on day 1 and paclitaxel intraperitoneally on day 8. Post-treatment group received 50 or 250 mg/kg XSLZJT orally after *i.p*. administration of 2 mg/kg paclitaxel. Subsequently, both groups received paclitaxel once every 2 days up to 4 times. Mechanical allodynia tests were performed on days 15 and 23. On day 23, the animals were sacrificed to harvest their L4-L6 vertebra for pathological examination. The results showed that, on day 15, pain sensitivity was significantly elevated in the control group (Figure 4B). When XSLJZT was administered before paclitaxel treatment, pain tolerance increased in the high-dose (250 mg/kg) group (Figure 5A). XSLZJT administered after paclitaxel treatment showed a high tolerance to mechanical pain stimulation on day 23 compared to the control group (Figure 5B). c-Fos is an immediate early gene that shows increased expression in neurons in response to various stimuli (Harris, 1998). We used c-Fos immunostaining to compare the expression of c-Fos in the spinal cord between the groups. The spinal cord of the blank group showed negligible c-Fos staining (Figure 6A), while the paclitaxel-only group showed significant c-Fos protein expression (Figure 6B). XSLJZT administered before or after paclitaxel treatment showed a dose-dependent reduction in c-fos expression compared to the control (Figures 6C, D). c-Fos expression did not differ significantly between the high-dose XSLJZT and blank groups. In conclusion, the results support that XSLJZT can attenuate paclitaxel-induced pain by modulating the neuronal response up to the spinal cord level.

### 3.5 Preventive effects of XSLJZT on paclitaxel-damaged DRG cells

**FIGURE 7:**
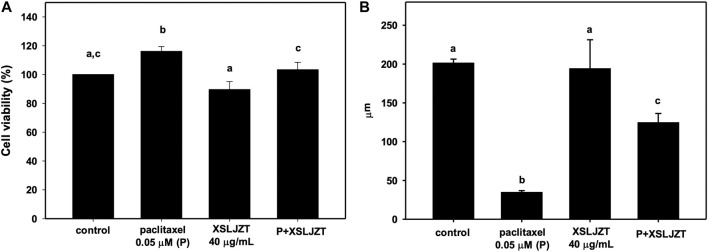
The cell viability of DRG neurons treated with 0.05 μM paclitaxel with or without XSLJZT for 24 h **(A)**. The neurite length of DRG neurons treated with 0.05 μM paclitaxel with or without XSLJZT for 24 h **(B)**. Results are presented as the mean ± S.D. The different alphabets show a significant (*p* < 0.05) difference between each other.

**FIGURE 8:**
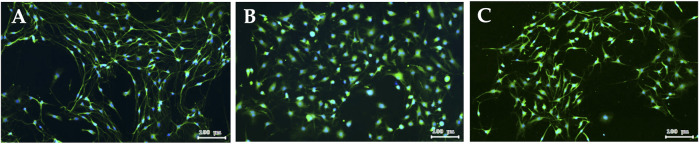
Fluorescence images of peripherin staining in DRG neuron. DRG neuron **(A)**; Paclitaxel induced nerve fibers shortening of DRG neuron for 24 h **(B)**; after 24 h co-culturing with XSLJZT, the medium was changed, and then co-cultured with 0.05 μM paclitaxel for another 24 h **(C)**.

DRG is a target for several pain control treatments because it can modulate peripheral and central sensory processing in conditions such as peripheral nerve injury and nociceptive stimuli. DRG treated with 0.05 μM paclitaxel for 24 h did not affect cell viability (Figure 7) or reduce DRG nerve fiber length (Figure 8). DRG co-culture with 40 μg/mL XSLJZT also did not show cell toxicity (Figure 7) and a deleterious effect on nerve fiber growth (Figure 8). Moreover, the medium was changed after a 24-h co-culture with the formulas. After performing a co-culture with 0.05 μM paclitaxel for another 24 h, the paclitaxel-induced nerve fiber shortening was reversed (Figure 8).

## 4 Discussion

According to our previous study (Lee et al., 2014), the most common prescriptions of the 115 breast cancer patient combined with TCM users were Jia Wei Xiao Yao San, Xiang Sha Liu Jun Zi Tang, and Gui Pi Tang. XSLJST showed, increase the spleen function, harmonize the stomach, and regulate the Qi flow to relieve pain. Therefore, we used three different animal experiments to determine whether the combination of XSLJST and paclitaxel would affect the effects of paclitaxel and whether it could actually improve the side effects caused by paclitaxel. Due to the use of different animal species and experimental design, the dosage of paclitaxel may be different in terms of efficacy and side effects. The doses of paclitaxel used to treat cancer, induce peripheral pain, and induce leukopenia were 20, 2, and 10 mg/kg. The dosage of XSLJZT was base on the clinical use. The clinical daily recommended dosage was 5 g, which was converted into 500 and 1,000 mg/kg for rats and mice respectively. However, we used the extract without adding excipients, and clinical excipients were half of the drug. Therefore, we adjusted the experimental dose to 250 mg/kg for rats and 500 mg/kg for mice according to the different animal species used in the experiment.

Seven out of the nine constituents of XSLJST isolated from *Atractylodes macrocephala* and *Wurfbainia longiligularis*, have been reported to exhibit antitumor effects against breast cancer (Lian et al., 2003; Kim et al., 2014; Wang et al., 2017; Jiang et al., 2018; Kim et al., 2018; Kong et al., 2019; Karatay et al., 2020). Two of the major compounds identified in XSLJZT, hesperidin and glycyrrhizic acid, have been reported to be effective against breast cancer cells (Lin et al., 2018; Kongtawelert et al., 2020). The cytotoxic effects of paclitaxel and XSLJZT were evaluated using *in vitro* and *in vivo* assays. At a high dose (5 mg/mL), XSLJZT inhibited P388-D1 cell proliferation. Although XSLJZT inhibited P388-D1 cells *in vitro*, it did not exhibit antitumor effects in P388-D1 CD2F1 tumor-bearing mice. Paclitaxel demonstrated inhibition of P388-D1 cells both *in vitro* and *in vivo*. In this study, co-administration with different dosages of XSLJZT slightly improved the survival rate of P388-D1 CD2F tumor-bearing mice, with an 11.1% increase in the lifespan compared to the group receiving paclitaxel alone. It was concluded that XSLJZT did not exert cytotoxic effects against P388-D1 cells *in vivo* without influencing the inhibitory effects of paclitaxel in P388-D1 CD2F tumor-bearing mice. In a meta-analysis, it was observed that patients receiving chemotherapy with a combination of herbal medicines had a higher survival rate than those receiving chemotherapy only.

Paclitaxel causes leukopenia in approximately 78%–100% of patients. Chemotherapy-associated myelosuppression commonly occurs after the first course of treatment; however, evidence also suggests that leukopenia may be associated with longer progression-free survival (Lee et al., 2011). Granulocyte-colony stimulating factor (G-CSF) is often used to stimulate the bone marrow to produce granulocytes and accelerate recovery from neutropenia-associated mortality following chemotherapy. In BALB/c-nu mice, paclitaxel treatment reduced the white cell count from day 2–3, until chemotherapy was discontinued for 5 days. In our study, XSLJZT administration before or after paclitaxel treatment restored paclitaxel-induced myelosuppression in BALB/c-nu mice, although the pre-treatment effect was more potent. However, when considering the overall clinical outcomes of patients undergoing paclitaxel, XSLJZT after treatment might benefit patients by assisting recovery from myelosuppression.

Chemotherapy-induced peripheral neuropathy (CIPN) is another frequently occurring condition that causes cancer patients to undergo suffering from the use of lifesaving therapies. Clinically, CINP is a symmetrically distributed sensory axonal neuropathy that starts in the fingers and toes and progresses toward the body. Paclitaxel-induced CINP is highly prevalent, dose-dependent, and usually treated by discontinuing the treatment. The mechanisms involved in this type of CINP include taxane-induced microtubule disruption that leads to Wallerian degeneration and neuroinflammation, resulting in atrophy of the DRG, hyperexcitability of peripheral neurons, axon degeneration, and secondary demyelination of peripheral nerves (Rowinsky and Donehower, 1995; Zajaczkowska et al., 2019). c-Jun and c-fos belong to a family of immediate early genes (Lee et al., 2001). In response to noxious stimuli, c-fos expression in the DRG is rapid, specific, and robotic. Therefore, immunohistochemical staining of c-fos expression in the dorsal horn neurons of the spinal cord is a frequently used marker to evaluate the analgesic effect of the tested compounds (Harris, 1998; Gao and Ji, 2009). In our study, XSLZJT effectively reduced pain and c-fos staining in the spinal cord. These results suggest that the reduced neuronal response in the spinal cord correlated with the reduced pain response in the experimental animals. The DRG is considered an active organ in the development of chronic pain and a clinical target for pain control because of its ability to modulate both peripheral and central sensory processing (Krames, 2014). In response to peripheral afferent fiber injury or inflammation, the DRG stimulates changes in glial cells, chemokines, cytokines, nerve growth factors, and ion channels. Studies have shown that paclitaxel induces degeneration of both the peripheral and central branches of DRG axons, nucleolar enlargement, and reduces neurite length (Jamieson et al., 2003; Tasnim et al., 2016). The paclitaxel dose used in our study was at the concentrations that had been demonstrated to have no effect on DRG neuron viability (Ustinova et al., 2013). XSLZJT restored the neurite shortening induced by paclitaxel, supporting the neuroprotective effect of this formula.

In addition to myelosuppression and neuropathy, chemotherapy induces many unexpected side effects, such as functional dyspepsia and diarrhea (Boussios et al., 2012). These gastrointestinal symptoms are compatible with Qi stagnation and spleen deficiency syndrome reported in traditional Chinese Medicine. XSLZJT deprives evil wetness and eliminates sputum while invigorating the spleen and replenishing Qi. Several studies have demonstrated that XSLZJT alleviates gastrointestinal symptoms clinically (Xiao et al., 2012; Shih et al., 2019).

**FIGURE 9:**
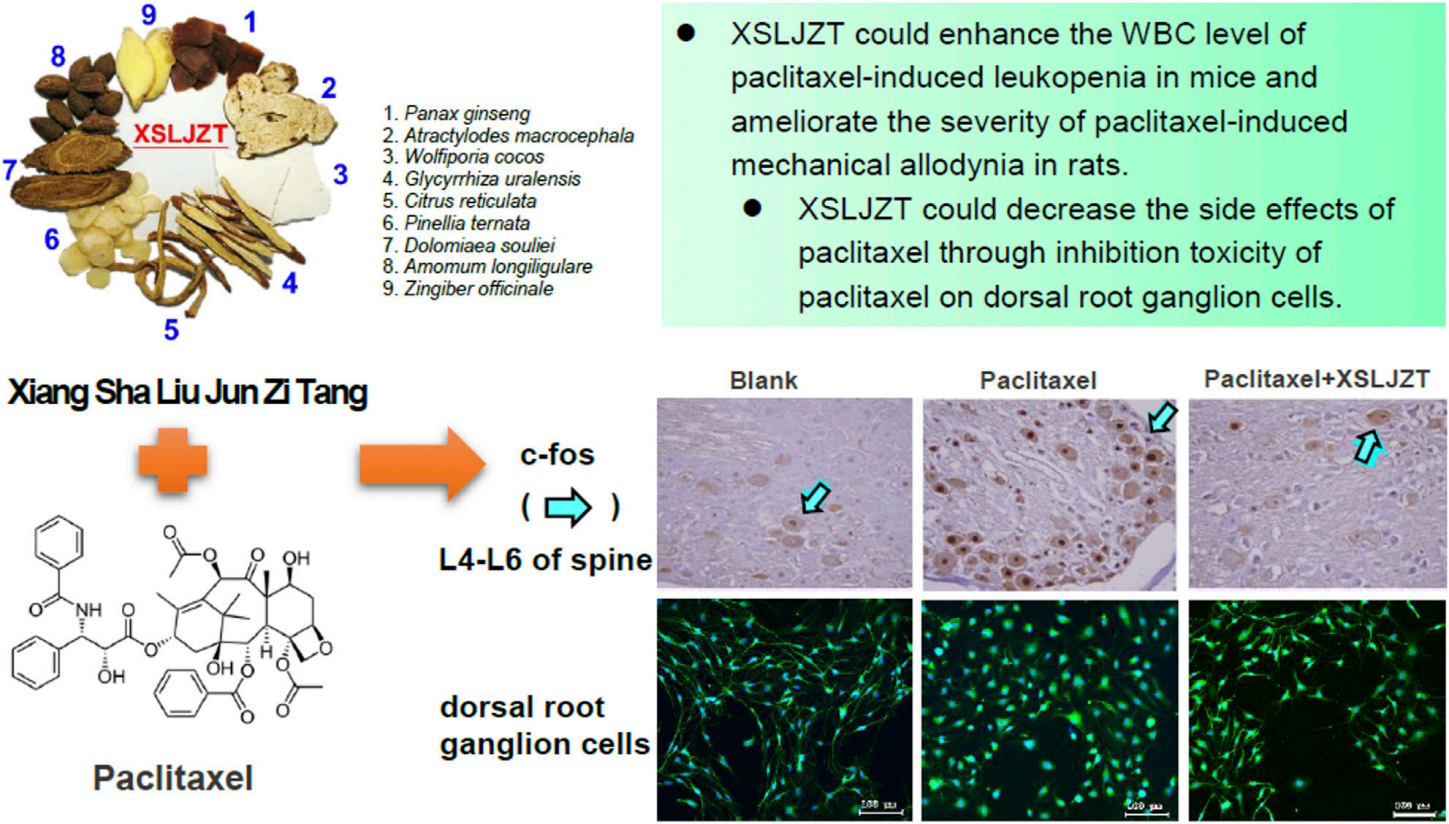
Summary of the XSLJZT on paclitaxel-induced leucopenia and neuropathy.

In conclusion, our study supports the finding that XSLZJT does not exert cytotoxic effects in P388-D1 CD2F tumor-bearing mice and does not significantly interfere with the therapeutic effects of paclitaxel. XSLJZT reduces paclitaxel-induced allodynia and immunosuppression. Based on our results, we suggest that XSLJZT could be prescribed after paclitaxel to reduce neurotoxic and leukopenia side effects and improve the patient’s quality of life (Figure 9).

## Data Availability

The original contributions presented in the study are included in the article/supplementary materials, further inquiries can be directed to the corresponding author.
